# Supplementary material for: Geochemical, Mineralogical and Morphological Characterisation of Road Dust and Associated Health Risks
Source: Int J Environ Res Public Health. 2020 Feb 28;17(5):1563. doi: 10.3390/ijerph17051563 (PMC7084894; doi:10.3390/ijerph17051563)
Supplement: Supplementary file 1 [file ijerph-17-01563-s001.pdf]

Supplemental Materials for

Geochemical, Mineralogical and Morphological Characterisation of Road Dust and Associated Health Risks

Carla Candeias[1], Estela Vicente[2], Mário Tomé[3], Fernando Rocha[1], Paula Ávila[4], Célia Alves[2*]

[1] Geobiosciences, Geotechnologies and Geoengineering Research Centre (GeoBioTec), Department of Geosciences, University of Aveiro, 3810-193 Aveiro, Portugal

[2] Centre for Environmental and Marine Studies (CESAM), Department of Environment, University of Aveiro, 3810-193 Aveiro, Portugal

[3] School of Technology and Management (ESTG), Polytechnic Institute of Viana do Castelo, Avenida do Atlântico, nº 644, 4900-348 Viana do Castelo, Portugal

[4] LNEG, National Laboratory of Energy and Geology, São Mamede de Infesta, Portugal

* Correspondence: celia.alves@ua.pt

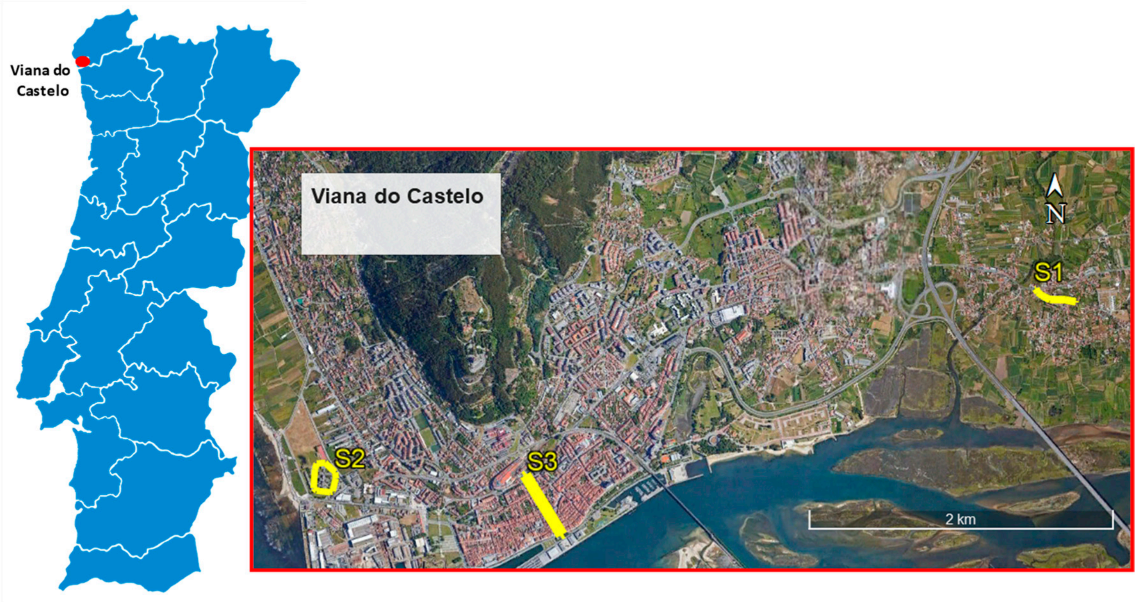

Fig. S1. Location of the sampling sites. Street 1 (S1) – suburban - Rua Alto Xisto, street with cobbled pavement made of granite cubes in a residential area; Street 2 (S2) - urban - road surrounding the campus of the Higher School of Technology and Management; Street 3 (S3) – urban - Avenida Combatentes da Grande Guerra, central artery connecting to the train station.

- **Parameters used in the calculations of sections 2.3 and 2.4**

Earth's crust individual elemental composition from Riemann and de Caritat [20]:

Al 77440 mg kg^{-1}; As 2 mg kg^{-1}; Ba 668 mg kg^{-1}; Br 1.6 mg kg^{-1}; Ca 29,450 mg kg^{-1}; Cl 640 mg kg^{-1}; Cr 35 mg kg^{-1}; Cu 14.3 mg kg^{-1}; F 611 mg kg^{-1}; Fe 30,890 mg g^{-1}; Ga 14 mg kg^{-1}; K 28,650 mg g^{-1}; Mg 13,510 mg kg^{-1}; Mn 527 mg kg^{-1}; Mo 1.4 mg kg^{-1}; Na 25,670 mg kg^{-1}; Ni 18.6 mg kg^{-1}; P 665 mg kg^{-1}; Pb 17 mg kg^{-1}; Rb 110 mg kg^{-1}; S 953 mg kg^{-1}; Sb 0.31 mg kg^{-1}; Si 30,3480 mg kg^{-1}; Sn 2.5 mg kg^{-1}; Sr 316 mg kg^{-1}; Ti 3117 mg kg^{-1}; V 53 mg kg^{-1}; W 1.4 mg kg^{-1}; Zn 52 mg g^{-1}; Zr 237 mg kg^{-1}.

Values of reference doses (R_fD_{route}), cancer slope factors (CSF), gastrointestinal absorption factors (ABS_{gi}) and inhalation unit risk (IUR) were taken from RAIS [24]:

R_fD_{route} - R_fD_{ing} (mg kg^{-1} d^{-1}) - As 3×10^{-4}; R_fD_{drm} - $RfD_{ing} \times ABS_{gi}$; R_fD_{inh} (mg m^{-3}) - As 1.5×10^{-5}

CSF - CSF_{ing} ((mg·kg^{-1} $d^{-1})^{-1}$) - As 1.50; Pb 8.5×10^{-3}; CSF_{drm} - CSF_{ing}/ABS_{gi}

ABS_{gi} - ABS_{gi} - As 1.00; Pb 1.00; ABS_{drm} - As 0.03

IUR - IUR ((µg $m^{-3})^{-1}$) - As 4.3×10^{-3}; Pb 1.2×10^{-5}

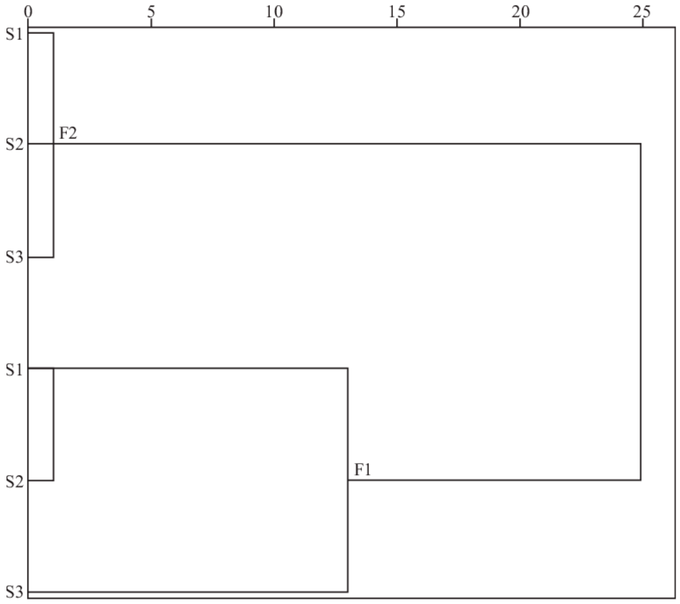

Fig. S2. Cluster analysis of the XRF results of road dust samples (S1 suburban environment influenced by agricultural activities; S2 and S3 urban streets).

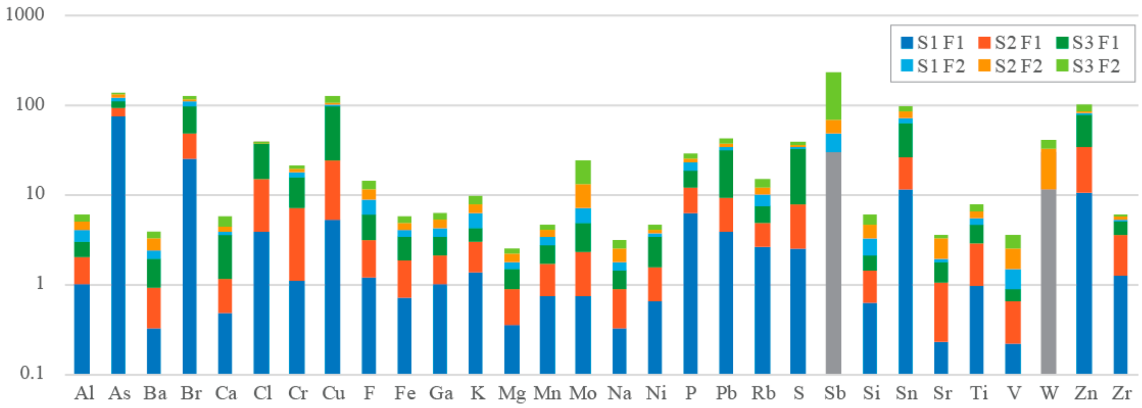

Fig. S3. Enrichment index for elements detected in road dust fractions (F1 – below 74 μm, F2 – between 74 μm and 1 mm). Below detection limit concentrations of Sb and W are represented in grey colour.
